# The *CD70*/*CD27* Axis in Tyrosine Kinase Inhibitor-Treated Chronic Phase Chronic Myeloid Leukemia Cells Is Not an Achilles Heel

**DOI:** 10.3390/hematolrep18040059

**Published:** 2026-08-17

**Authors:** Jennifer Cassels, Mark E. Drotar, Alyson MacNeil, Michael W. Moles, Ya-Ching Hsieh, Cassie J. Clarke, Eoghan Forde, Lorna Jackson, Moira A. Elliott, Julie Jacobs, Piotr Zabrocki, Mhairi Copland, Helen Wheadon, Alison M. Michie, Heather G. Jørgensen

**Affiliations:** 1 Paul O’Gorman Leukaemia Research Centre, School of Cancer Sciences, College of Medical, Veterinary and Life Sciences, University of Glasgow, 21 Shelley Road, Glasgow G12 0ZD, UK; jennifer.cassels@glasgow.ac.uk (J.C.); yaching.hsieh@gmail.com (Y.-C.H.); lorna.jackson.1@outlook.com (L.J.); mhairi.copland@glasgow.ac.uk (M.C.); helen.wheadon@glasgow.ac.uk (H.W.); alison.michie@glasgow.ac.uk (A.M.M.); 2 Centre for Healthcare Randomised Trials (CHaRT), Aberdeen Centre for Evaluation, Health Sciences Building, Foresterhill, Aberdeen AB25 2ZD, UK; 3 Scotland Institute, School of Cancer Sciences, College of Medical, Veterinary and Life Sciences, University of Glasgow, Switchback Road, Glasgow G61 1BD, UK; c.clarke@crukscotlandinstitute.ac.uk; 4 Medical Research Council Centre for Inflammation Research, Queen’s Medical Research Institute, 47 Little France Crescent, Edinburgh EH16 4TJ, UK; 5 Scottish National Blood Transfusion Service, Jack Copland Centre, Currie, Edinburgh EH14 4BE, UK; moira.elliott2@nhs.scot; 6 argenx BV, Industriepark Zwijnaarde 7, Zwijnaarde, 9052 Ghent, Belgium; jjacobs@argenx.com (J.J.); pzabrocki@argenx.com (P.Z.)

**Keywords:** leukemia stem cells, Wnt, signal blockade

## Abstract

**Background/Objectives**: Revolutionary small molecule, targeted, tyrosine kinase inhibitors (TKIs) in the clinic have engendered the perception that optimal treatment has already been established for diseases like chronic myeloid leukemia (CML). Addressing *BCR::ABL1* oncokinase domain mutations in CML is unlikely to redress disease recrudescence owing to persistent leukemia stem cells (LSCs). Therefore, it is necessary to consider an alternative strategic approach: disrupting LSC interactions with the protective microenvironment and/or immune system. The interaction of the TNF-α superfamily member, *CD27*, with its upregulated ligand, *CD70*, initiates survival signaling specifically in CML cells when *BCR::ABL1* is inhibited by TKIs and thus represents an attractive target. Previous studies modulating the expression of *CD27* on LSCs in a mouse model of advanced phase CML were encouraging. In our study, we explored the *CD70*/*CD27* axis as a therapeutic target in early-phase disease. **Methods**: Primitive *CD34*+ cells from treatment-naïve chronic phase (CP) CML patients were drug-exposed in an in vitro co-culture system; combination drug treatments of the therapeutic anti-*CD70* antibody and TKIs, nilotinib, were assessed in our CP CML murine model. **Results**: The blockade of the *CD70*/*CD27* axis in combination with TKIs did not result in greater LSC elimination than with nilotinib as a single agent in our CP CML models. Nonetheless, there was an observed reduction in *CD70*+ cells with combination treatment. **Conclusion**: Further preclinical study of the antibody as an adjunct in *CD70*-expressing hematological malignancy is perhaps warranted.

## 1. Introduction

Chronic myeloid leukemia (CML) is a relatively rare cancer with an annual incidence estimated at 1 in 100,000 in Western Europe. CML, being predominantly a disease of older age, significantly contributes to global morbidity and mortality, especially with the world’s aging population.

CML has a comparatively simple genetic landscape with the singular causative chromosomal aberration resulting from a reciprocal translocation between the long arms of chromosomes 9 and 22 ([t(9;22) (q34;q11)]) [1]. This event leads to the formation of the Philadelphia (Ph) chromosome and the *BCR::ABL1* fusion oncogene as a result of the c-ABL proto-oncogene from chromosome 9 transferring to a 5.8 kb major breakpoint cluster region (M-BCR) on chromosome 22 [2]. *BCR::ABL1* encodes a 210-kD phospho-oncoprotein that has constitutive tyrosine kinase activity that is uniquely necessary and sufficient for malignant transformation [3,4,5]. With advancing technologies, breakthroughs over the last couple of decades in the understanding of CML pathogenesis at a molecular level led to the development of molecularly targeted tyrosine kinase inhibitors (TKIs). The revolutionary introduction of TKIs into the clinic from the 2000s has perhaps engendered the perception that optimal treatment has already been established, but there are still many challenges to be faced in CML.

Hematopoietic stem cell transplantation (HSCT) prompted clinical interest in immunotherapy for CML in the 1970s as donor allogeneic T-cells were shown to exhibit a potent graft-versus-leukemia effect in comparison to syngeneic or T-cell-depleted grafts [6]. In recent years, it has become increasingly evident that the microenvironment and/or immune system may have a tumor-protective role. Therefore, a paradigm shift in the clinical approach to CML is now warranted to address the impact of leukemia stem cell (LSC) interactions. The development of increasingly potent TKIs is unlikely to redress the problems of resistance and recrudescence owing to the persistence of CML LSCs that are *BCR::ABL1*-kinase independent and therefore not killed by oncokinase inhibition [7,8].

*CD27* is a TNF-receptor superfamily member expressed on subgroups of B, T and natural killer (NK) cells and has been shown to have a role in differentiation and expansion of effector T-cells [9]. Using high-density arrays to compare gene expression in murine HSCs and their mature progeny, Wiesmann et al. [10] showed that *CD27* was also expressed by about 90% of purified hematopoietic stem cells (HSCs) from the bone marrow (BM). *CD27* signaling on HSCs and early progenitors has been shown to provide a negative feedback signal to leukocyte differentiation, especially B cells. Nolte et al. stimulated progenitor cells, expressing the *CD27* receptor, with its *CD70* ligand, resulting in the inhibition of in vitro colony formation and in vivo lymphocyte outgrowth [11]. *CD70* is a type II transmembrane glycoprotein and is a unique ligand for *CD27* [12]. Upon immune activation, *CD70* has been shown to be expressed on lymphocytes and subsets of dendritic cells [13].

Knowledge derived from studies in acute myeloid leukemia (AML) and CML [12,13,14] suggests that *CD70*/*CD27* signaling can confer pro-survival and proliferative advantages to hematological malignant cells. In CML, *BCR::ABL1*-driven stabilization of beta-catenin results in constitutive Wnt signaling; TKI activity represses miR-29c downstream of both Wnt and *BCR::ABL1* to directly upregulate SP1 while also downregulating DNMT1 through repression of miR-29a/b [13]. SP1 binds the promoter region of the *CD70* gene. Therefore, when TKIs are present, *CD70* promoter demethylation occurs in Ph+ CML cells, resulting in the availability of *CD27* signaling for survival. Schürch et al. investigated modulation of the immune system through *CD27* expression on LSCs in a mouse model of advanced-phase CML [12]. Nuclear localization of active beta-catenin and TRAF2 and NCK-interacting kinase (TNIK), consequent to binding of *CD27* by its ligand, *CD70*, and expression of Wnt target genes, resulted in differentiation and increased proliferation of murine LSCs. Furthermore, blocking of LSC *CD27* signaling prolonged survival.

In the present study, we explored the *CD70*/*CD27* axis as a therapeutic target in early-phase disease, the stage when most CML patients present clinically. We examined the cell autonomous expression levels of *CD70* and *CD27* of primary chronic phase (CP) CML cells cultured in suspension. We also co-cultured CML cells, using the *CD70*-expressing HS-5 stromal cells to in vitro model physiological microenvironmental crosstalk and to trigger or boost *CD70*/*CD27* signaling in the leukemia cells in the bicellular 2D model in the presence of TKI and/or *CD70*-blocking antibody to determine the effect of the combination on leukemia cell viability. Importantly, we also assessed the effect of the therapeutic blocking *CD70* antibody on LSC persistence in a CP CML mouse model [15], as interactions with not only the tumor BM microenvironment but also accessory cells of the immune system may be critical for pathophysiological presentation of *CD70* to its receptor, *CD27*.

## 2. Materials and Methods

### 2.1. Cell Lines and Primary Cell Isolation

Ethical approval for this study was obtained from the West of Scotland Research Ethics Committee, NHS Greater Glasgow and Clyde (United Kingdom; REC Reference 15_WS_0077). CML patients presenting in CP were consented to provide peripheral blood (PB) or leukapheresis samples from which stem/progenitor cells were enriched by positive magnetic selection (CliniMACS, Miltenyi Biotec, Bisley, UK) and cryopreserved at >90% *CD34*+ cells. Cryopreserved CML cells were recovered slowly from liquid nitrogen using a solution containing 100 U/mL DNase I (StemCell Technologies, Cambridge, UK), 20% (*v*/*v*) Human Serum Albumin (SNBTS, Glasgow, UK), 1 mM MgCl_2_ (Sigma-Aldrich, Gillingham, UK) and 115 mM trisodium citrate (Sigma-Aldrich) in 1× Dulbecco’s Phosphate Buffered Saline (DPBS) (Invitrogen, Paisley, UK). CML *CD34*+ cells were cultured in a growth factor-supplemented serum-free medium as previously described [8].

Human chronic myeloid leukemia (CML), K562, and KCL22 were purchased from the Leibniz Institute DSMZ—German Collection of Microorganisms and Cell Cultures GmbH, Braunschweig, Germany. The human stromal cell line, HS-5, was sourced from ATCC, Manassas, VA, USA.

### 2.2. Flow Cytometry Analysis

CML cells were stained at a concentration of 5 × 10^5^ cells/100 μL Hank’s Buffered Saline Solution (HBSS) in the presence of monoclonal antibodies directed against *CD34* (PerCP; clone: 8G12; BDBiosciences, Wokingham, UK), *CD27* (PE; clone: M-T271, BioLegend, London, UK), and *CD70* (APC; clone: 113-16, BioLegend), and fluorescent events were acquired on a FACSCanto II analyzer (BDBiosciences). For detection of apoptosis, 5 × 10^5^ cells/100 μL HBSS were stained with Annexin V (FITC) and 7-AAD (BDBiosciences). Data analysis was performed with FlowJo v9 analysis software (Tree Star, Inc., Ashland, OR, USA). To distinguish leukemia cells from co-culture cell lines, the harvested cells were surface stained with anti-CD45 before testing apoptosis as described.

### 2.3. RNA Isolation, Quantitative Real-Time Polymerase Chain Reaction (qRT-PCR), and In Silico Analysis of Publicly Available Datasets

RNA was purified using the RNeasy Mini Kit (Qiagen, Hilden, Germany) and reverse transcribed to cDNA with SuperScript III Reverse Transcriptase (Invitrogen) according to the manufacturer’s instructions. Gene expression was analyzed by quantitative real-time PCR performed in a 7900HT Fast Real-Time PCR system (ThermoFisher Scientific, Birmingham, UK) using TaqMan Gene Expression Assays (ThermoFisher Scientific) for the following targets: *CD27* molecule (*CD27*, Hs00386811m1), *CD70* molecule (*CD70*, Hs00174297m1) and glucuronidase β (GUSB, Hs00939627m1).

Expression of SP1, which, according to the literature, should be downregulated when *BCR::ABL1* or Wnt is active and conversely upregulated when *BCR::ABL1* is inhibited by TKIs, was analyzed in a publicly available dataset hosted on the Stemformatics interface [16]. The Affymetrix Gene ST platform comprises a large array design made up of exon-based probe sets that are mapped to gene transcripts but does not include mismatch probe sets that are normally used as negative controls for assessing chip effects. Instead, antigenomic probe sets are included on the chip as essentially negative control probes. There should not be any hybridizations of gene products to these antigenomic probes. The median of antigenomic probe set expression determined the expression threshold for the array dataset.

### 2.4. In Vitro Assessment of CD70 Blocking Antibody Treatment of CML Cells

*CD70*-expressing HS-5 human stromal cells were used as a feeding signal to stimulate *CD27* signaling in primary CML *CD34*+ cells rather than rely on auto-activation in leukemia cell monoculture. The use of a therapeutic antibody by blocking *CD70*/*CD27* engagement and, hence, downstream Wnt signaling in the *CD27*-expressing LSC would be expected to impact LSC growth. CML *CD34*+ cells were seeded at a density of 2 × 10^5^/mL on a confluent human stromal monolayer of HS-5 cells in growth-factor-supplemented serum-free medium [8], in the presence of an anti-human *CD70* blocking antibody (41D12 FcD; argenx BV, Gent, Belgium), over a time course and analyzed for apoptotic cell death by flow cytometry.

### 2.5. In Vivo Assessment of CD70 Blocking Antibody in a CP CML Mouse Model

All mice were maintained at the University of Glasgow Central Research Facilities under standard animal housing conditions in accordance with local and home-office regulations. The efficacy of anti-*CD70* alone and in combination with TKIs was assessed in the SCL-tTA/*BCR::ABL1* double transgenic (DTG) mouse model of CP CML, in which *BCR::ABL1* is inducibly expressed in HSC, resulting in leukemia that closely resembles the human disease [15]. To synchronize leukemia development, pooled BM from CD45.2 donors was transplanted into CD45.1 hosts, following lethal irradiation. Confirmation of leukemia induction, on removal of tetracycline, was determined by elevated total white blood cell count. Thereafter, mice were treated, or not, for 4 weeks with 50 mg/kg nilotinib once daily, reconstituted in the vehicle 10% N-methyl-2-pyrrolidinone in PEG300, by oral gavage (standard dosing, according to [17]), 10 mg/kg *CD70* blocking antibody (murine-specific clone FR-70 FcD; argenx BV) by IP injection every three days, or their combination. The FR-70 dosing schedule was adopted from [13]. At the end of the treatment period, a maximum of 6 mice from each cohort of 10 were sacrificed for immediate analysis, with the remainder followed for survival. The impact of the individual drugs and combinations on the survival of donor (*BCR::ABL1*+) Lin- Sca1+ c-kit+ (LSK), long-term HSCs and restricted progenitors (HPC1 and HPC2) was determined in PB, BM and spleen by flow cytometry as previously described [18]. Surface expression of *CD70*/27 on PB cells was analyzed post-mortem.

### 2.6. Statistical Methods

Ordinary one-way ANOVA was performed to compare the effect of an independent variable, such as drug treatment, on a dependent variable, such as cell surface protein expression. If the test revealed a statistically significant difference between at least two groups, Tukey’s honestly significant difference (HSD) post hoc test indicated where the differences lay across multiple comparisons.

## 3. Results

### 3.1. CML Cells Co-Express CD70/CD27 at Low Levels

**Figure 1:**
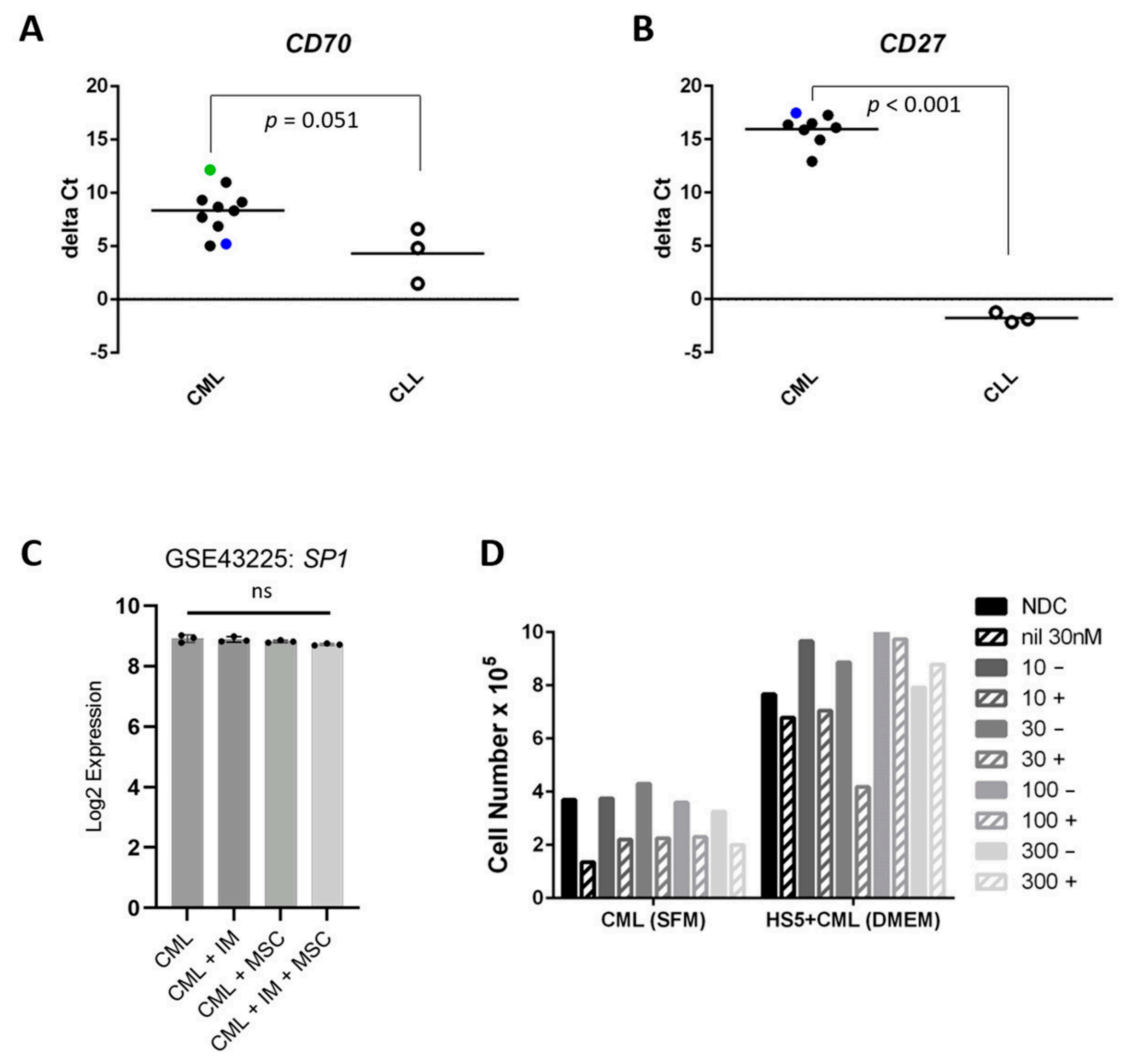
*CD70*-expressing HS-5 stromal cells support CML *CD34*+ cell growth and protect against TKIs, but an anti-*CD70* antibody does not synergize with nilotinib in vitro. (**A**) To elucidate the presence of the target for the therapeutic antibody, Q-PCR for *CD70* and its receptor *CD27* was performed in leukemia cells. Higher mean *CD70* mRNA expression was detected in CLL (open circles, *n* = 3 patients; positive control for expression) compared to CML *CD34*+ patient cells (black circles, *n* = 8 patients) or Ph+ human cell lines, K562 (blue circle) and KCL22 (green circle) using a relative quantification method whereby Ct (gene of interest) − Ct (reference gene) = ΔCt, so a negative value indicates relative higher expression. Unpaired student’s t-test performed for statistical significance. (**B**) Significantly higher *CD27* mRNA was detectable in CLL (positive control for expression) than CML patient cells. (**C**) In silico analysis of SP1 expression, a transcription factor known to be regulated by *BCR::ABL1* and/or Wnt that binds to the promoter region of the *CD70* gene, in an independent dataset generated from the culture of primary human CML *CD34*+ cells with or without MSC stromal support, with or without imatinib (IM) [19]. Inactivation of *BCR::ABL1* with TKIs was expected to upregulate SP1 [13]. (**D**) CML *CD34*+ cells grew better on *CD70*+ HS-5 MSC stromal support in HS-5 medium (HS5+CML (DMEM)) to mimic the BM microenvironment with respect to serum-free medium supplemented with physiological concentration growth factors (CML (SFM)). Stromal cells can offer protection against TKI killing [19]. Therefore, as predicted, CML *CD34*+ cells were less sensitive to a TKI (nilotinib; nil 30 nM) on *CD70*+ HS-5, but anti-*CD70* (titrated from 10 to 300 μg/mL, presence indicated by ‘+’) did not augment the response to nilotinib (*n* = 1). Feeder cells were unaffected by treatments. NDC: no drug control. ns: not significant.

To elucidate the presence of the target for the therapeutic anti-*CD70* antibody, Q-PCR for *CD70* and its receptor, *CD27*, as well as flow cytometric analysis of surface protein expression, was performed in leukemia cells. Ph+ cells, either CP CML *CD34*+ primary patient cells or human cell lines, expressed slightly lower levels of *CD70* by relative quantification than primary chronic lymphocytic leukemia (CLL) cells (positive expression control) at the gene level, and *CD27* was expressed at an even lower level, as indicated by a high positive ΔCt value (Figure 1A,B). This finding was corroborated by the presence of *CD70* protein on the surface of primary CML cells and a lack of detectable *CD27* surface protein expression. 

SP1 is a transcription factor known to be regulated by *BCR::ABL1* and/or Wnt that binds to the promoter region of the *CD70* gene. Inactivation of *BCR::ABL1* with TKIs is therefore expected to upregulate SP1. In an independent publicly available dataset generated from the culture of primary human CML *CD34*+ cells with or without mesenchymal stem cell (MSC) stromal support that protects against TKI killing, SP1 expression was calculated using the median of expression for the Gene ST array and compared against the median of above-threshold Log2 normalized probe expression values [19]. The detection threshold in the Zhang dataset was Log2 3.96; the overexpressed median was Log2 6.55 [19]. SP1 Log2 expression was in excess of eight and did not significantly differ between each scenario (plus/minus MSC, plus/minus imatinib) (Figure 1C).

### 3.2. Effect of Blocking CD70 in CML Cells In Vitro and In Vivo

In CML, the Wnt/β-catenin pathway that regulates stem cell self-renewal is constitutively active because of *BCR::ABL1*-mediated β-catenin stabilization [13]. Wnt signaling is inhibited by the miR-29 family (29a, 29b and 29c) [20]. miR-29b has been shown to downregulate DNA methyl transferase 1 (DNMT1) by targeting SP1, a known transactivating factor of DNMT1 [21]. The intention was to assay the on-target effects of blocking the *CD70* antibody alone or in combination with the second-generation TKI, nilotinib, at the molecular level in human CML *CD34*+ cells. In keeping with the lack of discernible biological effect (e.g., enhanced cell death) of blocking *CD70* antibody treatment plus TKIs in suspension culture in vitro, none of the molecular targets mentioned above were found to be modulated at the transcript level in our experiments. This result led us to consider in silico analysis of an independent dataset generated from the culture of primary human CML *CD34*+ cells with or without MSC stromal support, with or without first-generation TKIs, imatinib, that showed no modulation of SP1 under these in vitro conditions (Figure 1C) [19].

We reasoned there may be insufficient auto-activation of *CD70*/*CD27* in suspension culture of CML *CD34*+ cells, as both *CD70* and *CD27* were expressed at low levels (Figure 1A,B). Therefore, CML *CD34*+ cells were cultured on *CD70*-expressing human stromal cell line, HS-5 (Figure 1D), to potentially activate trace *CD27* on CML *CD34*+ cells through interaction with its ligand on the accessory (stromal) cells, in the presence of 30 nM nilotinib (approaching IC50 in suspension culture and previously demonstrated to inhibit *BCR::ABL1* signaling without inducing excess cell death so combination effect(s) could be seen [22]) and increasing concentration of anti-*CD70* antibody (10–300 μg/mL). This approach neither initiated non-canonical Wnt signaling activity, as indicated by a lack of modulation of target genes, nor improved CML *CD34*+ cell killing over and above nilotinib alone (Figure 1D). Notably, the CML *CD34*+ cells were more viable and proliferative on HS-5 compared to suspension culture, which suggested that local physical interactions and/or paracrine growth factor signaling were at play to counter the effects of *BCR::ABL1* silencing by nilotinib. Therefore, we focused on the in vivo model of CP CML in which the accessory cells of the immune system could present *CD70* to *BCR::ABL1*+ *CD27*+ LSCs in their natural environment.

**Figure 2:**
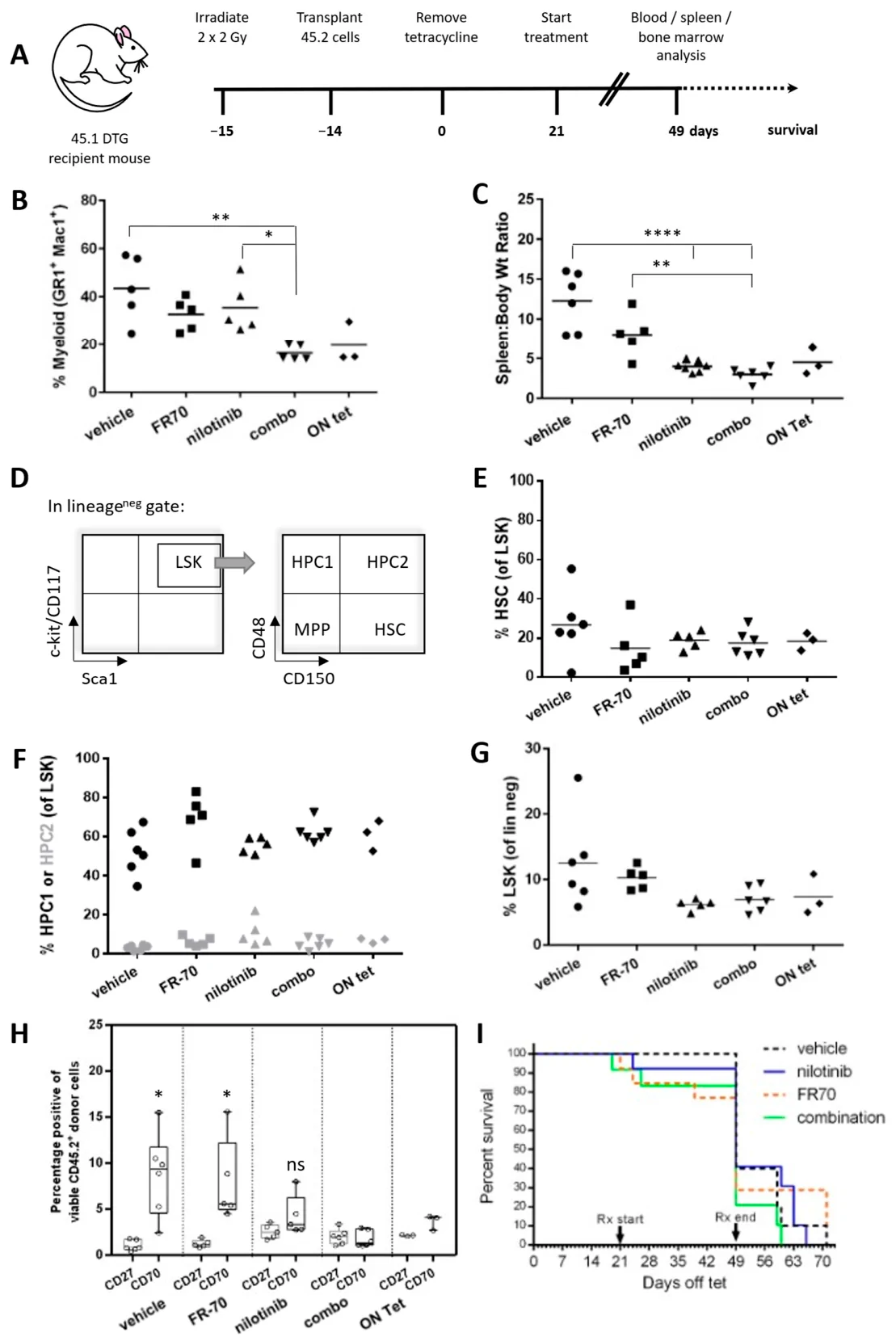
A drug feeding experiment in a double transgenic (DTG) chronic phase (CP) CML mouse model showed no combined effect of anti-*CD70* with nilotinib on stem/progenitor cell compartment subpopulations: (**A**) Schematic diagram of the transplantation and dosing schedule of the SCL-tTA/*BCR::ABL1* DTG mouse model. In the absence of tetracycline, the tetracycline-controlled transactivator (tTA) protein binds the TRE promoter, hence inducibly expressing *BCR::ABL1*. The stem cell leukemia (SCL) promoter exclusively expressed in hematopoietic cells enables *BCR::ABL1* expression in HSCs and progenitors, resulting in leukemia that closely resembles the human disease. Treatment with nilotinib (50 mg/kg) daily by oral gavage and/or FR-70 (10 mg/kg) every three days by intraperitoneal injection or vehicle (PBS) was begun 21 days after removal of tetracycline from the drinking water. (**B**) A myeloproliferative response as shown by the high percentage of *Gr1*+ *Mac1*+ cells in the PB of vehicle-dosed animals was observed, which was reverted by the combination (‘combo’) of nilotinib and FR70 (murine-specific anti-*CD70*) to normal values of the transplant controls kept on tetracycline (‘ON Tet’). (**C**) Splenomegaly of the vehicle-dosed animals was evident with respect to nilotinib and the combination. (**D**) Gating strategy to resolve distinct immature hematopoietic subpopulations: LSK; HPC1/2, hematopoietic progenitor cell; MPP, multipotent progenitor; HSC, hematopoietic stem cell. Using SLAM markers to resolve distinct subpopulations of (**E**) *CD150*+ *CD48*− (HSC), (**F**) *CD150*− *CD48*+ (HPC1) and *CD150*+ *CD48*+ (HPC2), and (**G**) bulk LSK cells did not reveal any greater effect of the combination with respect to nilotinib as a single agent in these hematopoietic compartments. (**H**) Surface protein expression analysis by flow cytometry on *CD45.2*+ donor bone marrow cells from primary host animals culled at their censor point showed a significantly higher percentage of *CD70*+ cells in the vehicle and FR70 groups but not the nilotinib monotherapy with respect to the combination (‘combo’) treatment. The percentage of viable *CD45.2*+ *CD27*+ donor cells in the nilotinib monotherapy treatment group was only significantly greater than the vehicle, not the combination. Ordinary one-way analysis of variance (ANOVA) was performed to reveal any statistical significance between at least two groups; Tukey’s HSD post hoc test revealed the pair-wise differences. * *p* < 0.05; ** *p* < 0.01; **** *p* < 0.001, ns not significant. ON Tet was not included in the ANOVA table. (**I**) Animals from each cohort were followed through treatment from day 21 (‘Rx start’ indicated on *Days off tet* axis by arrow) and after stopping treatment at day 49 (‘Rx end’) until signs of morbidity (e.g., sustained weight loss) precipitated the decision to cull.

Using the SCL-tTA/*BCR::ABL1* double transgenic (DTG) mouse model of CP CML [15], in the absence of tetracycline, the tetracycline-controlled transactivator (tTA) protein binds the TRE promoter, hence inducibly expressing *BCR::ABL1*. The stem cell leukemia (SCL) promoter exclusively expressed in hematopoietic cells enables *BCR::ABL1* expression in HSC and progenitors, resulting in leukemia approximately 3 weeks later that closely resembles the human disease (Figure 2A). The expected myeloproliferative response to the removal of tetracycline from the drinking water for 21 days prior to initiating treatment of DTG mice was confirmed by the high percentage of Gr1+ Mac1+ cells in the PB of vehicle-dosed animals. Myelocytosis was reverted by the FR-70 plus nilotinib combination (‘combo’) to a value lower than nilotinib monotherapy and the transplant control arm (‘ON Tet’) (Figure 2B; *p* < 0.05). Spleen cellularity across the treatment arms did not differ; however, splenomegaly of the vehicle-dosed animals was evident from the spleen-to-body weight ratio with respect to nilotinib and the combination (Figure 2C; *p* < 0.001). Using SLAM markers (CD150, CD48) (Figure 2D) to resolve distinct subpopulations within the LSK cells of the BM, the HSC (150+ 48−; Figure 2E) and HPC1 (150− 48+) or HPC2 (150+ 48+; Figure 2F) were not different across arms in this study. The bulk LSK population did not reveal any greater effect of the combination with respect to single agents (Figure 2G).

Secondary transplants may have revealed differences in leukemic re-populating potential between the treatment arms. Ordinary one-way ANOVA was performed to compare the effect of drug treatments on *CD27* or *CD70* surface protein expression. The test revealed that there was a statistically significant difference between at least two groups in *CD27* (F(3,18) = 5.985, *p* = 0.0052) and *CD70* (F(3,18) = 5.331, *p* = 0.0083). Tukey’s HSD test for multiple comparisons found that *CD70* surface protein expression on CD45.2+ donor cells from primary host animals culled at their censor point showed significantly lower frequency with the combination treatment (average ± standard deviation; 1.77 ± 0.81%) than was seen with either FR70 antibody (7.99 ± 4.08%; *p* = 0.033, 95% C.I.= 0.4109 to 12.04 ), or vehicle (8.74 ± 4.12%; *p* = 0.011, C.I. = 1.431 to 12.52) (Figure 2H). Nilotinib monotherapy (4.28 ± 1.96%) *CD70* frequency was numerically greater than combination treatment but did not reach statistical significance (Figure 2H). Further, the mean percentage of viable CD45.2+ donor cells that were *CD27* positive with nilotinib monotherapy (2.57 ± 0.68%) was numerically greater than the combination (2.02 ± 0.75%), although not statistically significant (Figure 2H). Average *CD27* frequency with nilotinib treatment was significantly greater than vehicle (1.04 ± 0.52%; *p* = 0.007, 95% C.I.= −2.671 to −0.3861; Figure 2H) suggesting possible accumulation or persistence of LSC-containing subpopulations, which we have previously ascribed to TKI activity [7,8].

Survival curves were generated for all arms of this study; a maximum of four animals from each cohort of 10 were followed on cessation of therapy at 49 days until the animals reached a humane endpoint (Figure 2I). No differences were noted between the experimental arms.

## 4. Discussion

This study aimed to propose a paradigm shift in the clinical approach to tyrosine kinase-driven stem cell diseases such as CML to address LSC interactions with the protective microenvironment and/or immune system rather than tumor bulk or cell-intrinsic properties of LSCs. The mainstay of treatment in CML remains oncoprotein-targeted TKI monotherapy, such as nilotinib. There are key areas of unmet clinical need in CML currently: CML LSC persistence despite inhibition of *BCR::ABL1* signaling and the linked concept of inability to confidently attempt treatment-free remission for all; frank TKI resistance through point mutation where TKIs become ineffective; and progression to blast crisis where TKI monotherapy does not achieve disease control. TKIs are very well tolerated. Most commercial drug development programs, therefore, aim to combat TKI resistance by addressing *BCR::ABL1* kinase domain mutations. However, the development of TKIs of increasing potencies is unlikely to redress the problems of resistance and disease recrudescence, owing to persistent LSCs that are *BCR::ABL1*-kinase independent and, therefore, not killed by TK inhibition. Indeed, *BCR::ABL1* inhibition may invoke survival mechanisms such as *CD70*/27 signaling to activate Wnt. Hence an alternative strategy and/or complementary target must still be sought.

ARGX-110 (also known as JNJ-74494550 or cusatuzumab) is a defucosylated monoclonal IgG1 antibody capable of targeting and neutralizing the human TNFα-like ligand, *CD70*. Fully humanized, it is reported that this therapeutic antibody can lyse *CD70*+ tumor cells via complement-mediated cytotoxicity (CDC), antibody-dependent cellular phagocytosis (ADCP) or enhanced antibody-directed cellular cytotoxicity (ADCC) [23,24]. Interim results from a Phase 1b clinical trial of ARGX-110 as a single agent conducted in nine patients with *CD70*+ T-cell lymphoma (TCL) reported four treated patients with signs of clinical/biological anti-tumor activity, two of which had >90% reduction in the circulating malignant clone (Table 1; ClinicalTrials.gov Identifier: NCT01813539/EudraCT 2012-005046-38) [25]. The antibody’s direct tumor lysis mechanism of action through blockade of *CD70* was attractive to test against *CD70*-expressing leukemia cells, such as CML. Our study aimed to address CML LSC persistence despite successful inhibition of *BCR::ABL1* signaling by TKIs by selectively targeting the *CD70*/*CD27* pathway that had previously been shown to be upregulated in CML cells upon *BCR::ABL1*-kinase inhibition and contributes to CML LSC survival [12,13]. However, in liquid suspension monoculture, we did not observe any direct tumor cell killing mediated through antibody binding to *CD70*+ primary CML *CD34*+ leukemia cells in response to the therapeutic antibody alone or in the presence of TKIs. Critically, the response of rare LSCs specifically and differentially marked from normal stem cells by *CD26* [26], *CD93* [27] or *IL1-RAP* [28], for example, and that comprise a small but significant proportion of the *CD34*+*CD38*- subpopulation that is presumed responsible for CML LSC persistence, likely was masked; fluorescence activated cell sorting of such LSCs from bulk *CD34*+ cells based on these putative markers is warranted but would have yielded too few cells to analyze. We attempted to improve activation of *CD70*/*CD27* signaling in the LSC fraction through presentation of *CD70* by HS-5 stromal cells to any CML *CD34*+ *CD27*+ cells contained in the co-culture, but the strategy did not improve efficacy; that may be explained by the low *CD27* expression levels on CML *CD34*+ cells. Together with our suspicion that ‘auto-activation’ through unicellular co-expression of *CD70* and *CD27* may not be optimal and to overcome the limitations of non-physiological two-cell, 2D co-culture in vitro models, we opted to test the combination in an in vivo model with a competent immune system to encourage possible ADCP to occur. Preclinical data in an in vivo model of CML had suggested a potential breakthrough in dual targeting of *BCR::ABL1* with TKIs and *CD70*/*CD27* signaling with ARGX-110 in a transplant model of more advanced phase disease [13].

In their murine BM transduction model of CML, Schürch et al. showed that *CD27* is expressed on murine *BCR::ABL1*+ leukemia stem/progenitor cells [12]. The binding of *CD27* to its ligand, *CD70*, induced overexpression of Wnt/β-catenin target genes, leading to increased proliferation of *BCR::ABL1*+ LSCs. Furthermore, blocking *CD70*/*CD27* signaling in LSCs resulted in delayed disease progression and prolonged survival of treated mice. Supporting data in *CD27*−/− mice showed improved survival. Important and encouraging data using primary human CML cells were generated in xenograft mice whereby recipients were treated within 1 week of transplant, which notably meant that short-term as opposed to long-term LSCs were studied [13]. However, the levels of engraftment and the clinical parameters displayed suggested that the human donor cells were from the more advanced phase of disease. Owing to low levels of engraftment by early CP CML cells (typically <5%), transplanted mice do not normally have overt signs and symptoms, e.g., leukocytosis and splenomegaly of CML, as observed by Riether et al. [13].

## 5. Conclusions

The blockade of the *CD70*/*CD27* axis, a hypothesized survival signaling pathway in CML *CD34*+ cells when *BCR::ABL1* signaling is inhibited by TKIs, did not result in greater LSC elimination than nilotinib as a single agent in our model of CP CML. Hence, further work is required to determine if a synergistic dose of both nilotinib and a blocking *CD70* antibody for in vivo use could be found; thereafter toxicity to normal *CD34*+ cells should be assessed. Owing to negative findings in our in vivo model, the latter control toxicity experiment was not justifiable in this study, but non-specific toxicity was not observed. Although we could not demonstrate an overt therapeutic advantage to combining anti-*CD70* with nilotinib over TKI monotherapy, we did see a reduction in *CD70*+ hematological cells in combination-treated animals. A fuller investigation of the putative role for the anti-*CD70* molecule as an adjunct in overtly *CD70*-expressing chronic hematological malignancy, such as chronic lymphocytic leukemia, may be warranted.

## Figures and Tables

**Table 1:** Clinical trials with therapeutic anti-*CD70* antibody, cusatuzumab.

Study Title	Trial No.	Drug Combination Tested	Patient No.	Phase	Reference/Notes
A Study of ARGX-110 in Participants with Advanced Malignancies	NCT01813539	cusatuzumab	99	I/II	Silence et al., 2013. doi: 10.4161/mabs27398 [23]
A Study of ARGX-110 in Combination with Azacytidine in Participants with Newly Diagnosed AML or High-Risk MDS	NCT03030612	azacitidine + cusatuzumab	38	I/II	Riether et al., 2020. doi: 10.1038/s41591-020-0910-8 [29]
A Study of Cusatuzumab Plus Azacitidine in Participants with Newly Diagnosed AML Who Are Not Candidates for Intensive Chemotherapy (CULMINATE)	NCT04023526	azacitidine + cusatuzumab	103	II	Pabst et al., 2023. doi:10.1016/S2352-3026(23)00207-7 [30]
A Study of Cusatuzumab Plus Azacitidine in Japanese Participants with Newly Diagnosed AML or High-Risk MDS Who Are Not Candidates for Intensive Treatment	NCT04241549	azacitidine + cusatuzumab	9	I	Ikezoe et al. 2022. doi: 10.1111/cas.15663 [31]
Cusatuzumab in Combination with Background Therapy for the Treatment of Participants with AML (ELEVATE)	NCT04150887	azacitidine + cusatuzumab + venetoclax	61	I	Active, not recruiting. Estimated study completion May 2027

Footnotes: cusatuzumab is the International Non-proprietary Name (INN) for the therapeutic anti-*CD70* antibody also known as ARGX-110 or JNJ-74494550. Abbreviations: AML, acute myeloid leukemia; MDS, myelodysplastic syndrome.

## Data Availability

Data is contained within the article.

## Abbreviations

The following abbreviations are used in this manuscript:

AML	Acute myeloid leukemia
BM	Bone marrow
CLL	Chronic lymphocytic leukemia
CML	Chronic myeloid leukemia
CMML	Chronic myelomonocytic leukemia
CP	Chronic phase
DNMT1	DNA methyltransferase 1
DTG	Double transgenic
HPC	Hematopoietic progenitor cell
HSC	Hematopoietic stem cell
HSCT	Hematopoietic stem cell transplant
LSC	Leukemia stem cell
LSK	Lineage Sca1 c-kit
MDS	Myelodysplastic syndrome
MPP	Multipotent progenitor
MSC	Mesenchymal stem cell
PB	Peripheral blood
Ph	Philadelphia chromosome
TCL	T-cell lymphoma
TKI	Tyrosine kinase inhibitor
TNFα	Tumor necrosis factor alpha
TNIK	TRAF2- and NCK-interacting kinase
